# ‘Staying Hot’: Investigating the influence of overnight conditions on the penile skin temperature during male sexual arousal—A novel methodology for nocturnal erection detection

**DOI:** 10.1002/bco2.328

**Published:** 2024-02-22

**Authors:** Hille J. Torenvlied, Evelien Trip, Wouter Olthuis, Loes I. Segerink, Jack J. H. Beck

**Affiliations:** ^1^ Department of Urology St. Antonius Ziekenhuis Nieuwegein Netherlands; ^2^ Faculty of Electrical Engineering, Mathematics and Computer Science, BIOS Lab on a Chip group Universiteit Twente Enschede Netherlands; ^3^ Department of Urology Leiden Universitair Medisch Centrum Leiden Netherlands

**Keywords:** erectile dysfunction, nocturnal erections, penile temperature, RigiScan, sexual arousal, temperature sensing

## Abstract

**Objective:**

The objective of this study is to assess the impact of overnight environmental conditions on erectile penile temperature within a controlled setting, with the aim of investigating the feasibility of using temperature measurements for nocturnal erection detection in erectile dysfunction diagnostics.

**Subjects/patients and methods:**

We conducted a proof‐of‐concept study involving 10 healthy male participants aged 20 to 25. The study was carried out at the Department of Urology, St. Antonius Ziekenhuis, the Netherlands. Penile temperature thermistor measurements were taken during visually aroused erections of participants in naked state and in simulated overnight condition (underwear and blankets). Main outcome variables were peak and baseline temperature during erectile periods. To minimize the impact of differences in erectile strength and duration between consecutive measurements, we applied randomization to the order of the environmental conditions.

**Results:**

We observed a significant increase in penile temperature during erection in both the naked (*p* < 0.01) and simulated overnight condition (*p* < 0.01). The mean temperature increase was 1.70 and 0.67*°*C, respectively. While penile temperature returned to baseline immediately after naked erections, the ‘Staying Hot effect’ was noted in the simulated overnight condition measurements, where the temperature remained elevated at peak temperature for the entire 30‐min period following the erection.

**Conclusions:**

The findings from this study indicate that the penile temperature not only significantly increases during naked sexual arousal but is also detectable under simulated overnight conditions. This underscores the potential of using temperature measurements for nocturnal erection detection, representing a crucial initial step in developing a modernized, non‐invasive sensor system for ambulatory erectile dysfunction diagnostics. Further research, including an overnight study, is needed to gain insights into the feasibility of utilizing penile temperature measurements for nocturnal erection detection and to assess the impact of the ‘Staying Hot effect’ on subsequent erection detection.

## 1 INTRODUCTION

The established gold standard for non‐invasive nature differentiation of erectile dysfunction (ED) is nocturnal erection detection using the RigiScan device.^1^ However, this system has largely fallen out of use in many healthcare settings due to outdated hardware and software leading to uncomfortable measurements and questionable validity.^2^, ^3^ Consequently, there is an urgent demand for a modernized and patient‐friendly ED diagnostic system. In the pursuit of advancing ED diagnostics, the question has arisen regarding the feasibility of using penile temperature measurements for nocturnal erection detection. Such an approach, devoid of a pressure component, is anticipated to enhance the patient experience and, consequently, diagnostic accuracy.^4^ Surprisingly, no prior studies have explored the potential of nocturnal erection detection through penile temperature sensing.

Several decades ago, researchers identified a significant increase in penile temperature during visually aroused erections in healthy men. This temperature rise is a result of the corpora cavernosa filling with warm, oxygenated blood, which leads to the conduction of heat to the adjacent penile skin. Subsequent investigations demonstrated a consistent correlation between vasocongestion, penile temperature, penile circumference and subjective arousal in this population.^5^, ^6^, ^7^, ^8^, ^9^, ^10^ However, these studies were conducted on naked men, who obtained visually or sexually aroused erections. It is reasonable to assume that thermal insulation provided by blankets and clothing may mitigate temperature fluctuations during nocturnal erections, potentially resulting in prolonged elevation of penile temperature post‐erection. The implications of these environmental factors on the feasibility of nocturnal erections remain unclear.

To predict the diagnostic utility of the patient‐friendly temperature‐based methodology and ensure the accurate interpretation of future overnight studies, it is imperative to investigate the impact of environmental conditions on penile temperature during erections within a controlled research framework. The present ‘Staying Hot’ study aims to assess the effects of simulated overnight conditions on erection detection using the penile temperature methodology, employing a study design aligned with existing literature.

## 2 SUBJECTS, MATERIALS AND METHODS

### 2.1 Study setting

The ‘Staying Hot’ study was conducted at the Department of Urology at the St. Antonius Ziekenhuis Nieuwegein, the Netherlands. The study received approval from the Medical research Ethics Committees United in Nieuwegein, the Netherlands on 22 February 2022 (NL79920.100.21, R21.114). The clinical trial was pre‐registered at ClinicalTrials.gov before enrolling test subjects (NCT05183581).

### 2.2 Participants

Ten healthy male participants aged 20–25 were enrolled between April and June 2022. The sample size was determined based on existing literature regarding penile temperature changes during visually aroused erections.^8^ All participants provided informed consent and received financial compensation for their participation. Exclusion criteria encompassed unwillingness to sign informed consent, an International Index of Erectile Function (IIEF)‐5 score below 22, a medical history of sickle cell anaemia, atherosclerosis, diabetes or usage of benzodiazepines.^11^ Participants were instructed to conduct the measurements alone in bed during the ambulatory measurements and to abstain from alcohol, sexual activity or masturbation prior to participation.

### 2.3 Materials and methods

**FIGURE 1:**
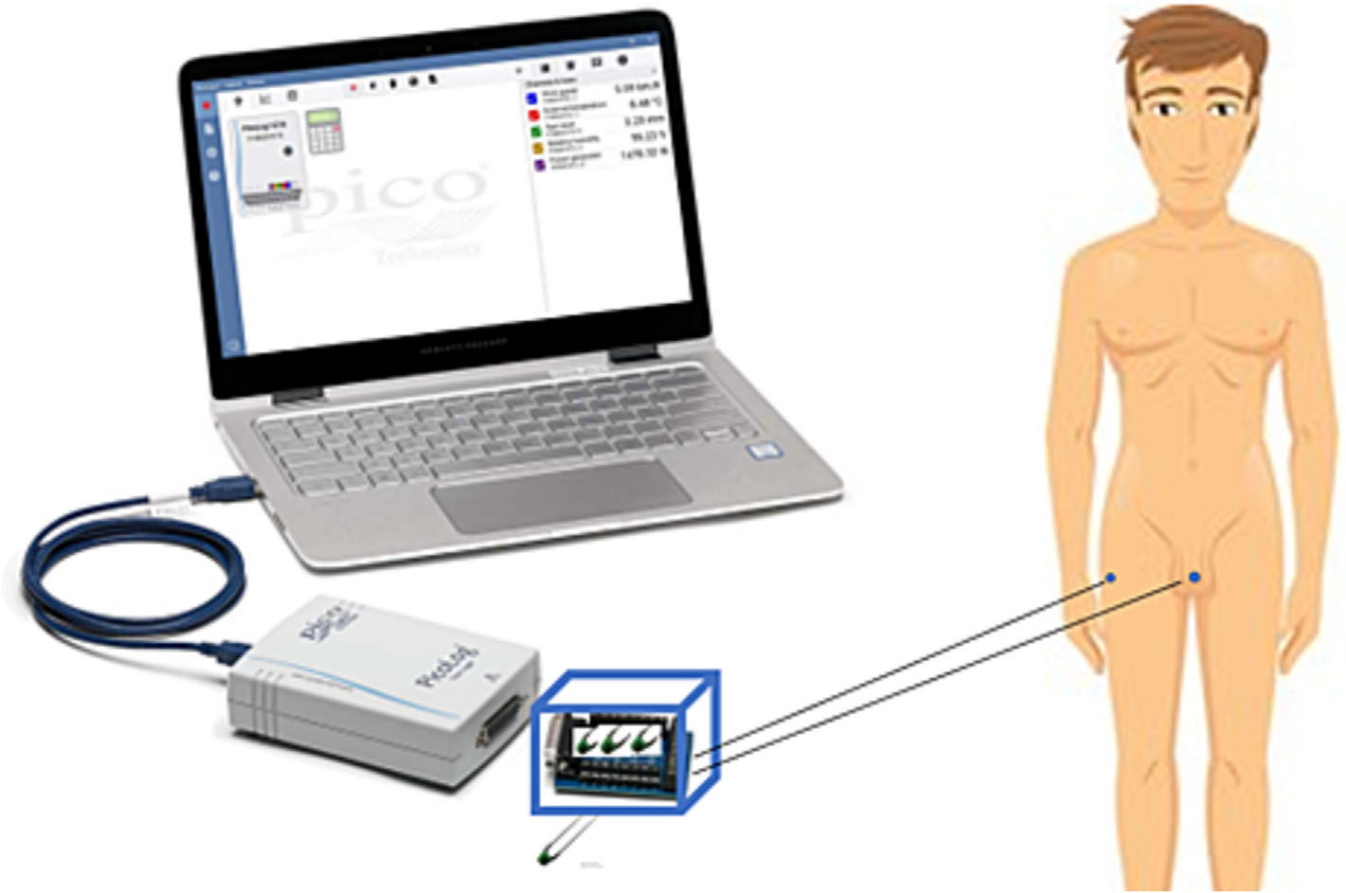
Schematic overview of the study set‐up for the ‘Staying Hot’ study consisting of two Ohmeda 3P T3312 temperature probes (Shenzhen Medke Technology Co., Shenzhen, Guangdong, China) combined through a terminal board to the Picolog 1216 data logger (Pico Technology, Cambridgeshire, UK).

Penile temperature was measured using a sensor system comprising Ohmeda 3P T3312 temperature probes (Shenzhen Medke Technology Co., Shenzhen, Guangdong, China).^12^ Given the non‐uniform nature of penile temperature in the flaccid state, it was imperative to maintain a consistent region of interest for our measurements. This necessitated the use of a contact sensor. We opted for thermistor sensors, specifically the Ohmeda 3P T3312 probes, which possess several advantageous attributes, including a compact 6 mm diameter, high precision, good sensitivity and cost‐effectiveness. To facilitate data acquisition, the temperature sensors were connected to a PicoLog 1216 data logger (Pico Technology, Cambridgeshire, UK) and a standalone Lenovo ThinkBook 13s‐IWL laptop.^13^ An electrical circuit with three 10 kΩ pull‐up/pull‐down OT360‐C78 resistors (Otronic, Cruquius, the Netherlands) was employed to ensure accurate conversion of output voltage values into temperature data. The data logging system was positioned near the participants' beds to facilitate unrestricted movement, minimize potential entanglement hazards and prevent system‐generated heat from affecting the temperature measurements. Figure 1 shows a schematic overview of the measurement system.

The placement of the temperature sensors followed a specific test design, with the first sensor situated on the dorsal penile skin proximal to the glans. Despite previous clinical research indicating that the highest temperature increase during an erection occurs in the glans itself, this proximal placement took into consideration factors such as the presence of the foreskin, varying circumcision status among subjects, and the influence of corpus spongiosum perfusion of the glans.^14^ The penile sensor was affixed to the skin using Leukosan 12 × 100 mm strips. Sensor placement was performed by the participants to ensure their privacy.

During the erection measurements, a second thermistor sensor was used for annotating the visually aroused erections. The test subjects held the temperature sensor in the palm of their hand, resulting in a noticeable increase in the sensor's temperature, signifying the initiation and conclusion of erections. This method ensured precise annotation of erection timing by the test subjects. The test subjects initiated measurements using the PicoScope 6 software. Subsequently, participants turned off the systems and removed all sensors. Measurements were considered successful if the duration of an erection lasted at least 5 min.

### 2.4 Study procedure

Each participant attained two visually aroused erections while being exposed to commercially available pornographic material. Masturbation was prohibited to prevent temperature increase due to friction energy. One erection measurement was conducted while naked in bed, and the other was performed with blankets and cotton underwear (referred to as ‘blanket measurement’). Given the absence of prior research on differences in Erection Hardness Scale between two consecutive visually aroused erections, a randomization process was employed to determine the measurement order (see Table 1). Group A started with the naked measurement, while Group B began with the blanket measurement.

**TABLE 1:** Outcome of the randomization.

Group	Measurement order	Test subjects
A	Naked–blanket	1, 3, 4, 6, 10
B	Blanket–naked	2, 5, 7, 8, 9

Each measurement comprised three phases: the baseline phase, erectile phase and cooling phase. Earlier studies have demonstrated that a bodily temperature increase of 2–3*°*C under blankets can be achieved within 10 min.^14^ Consequently, the duration of the baseline measurement was set at 10 min. During the erectile phase, subjects were instructed to attain a visually aroused erection and sustain it for a duration of desirably 10 min. Previous studies have determined this duration as the time required to reach maximum penile tumescence and temperature during an erection.^8^ Erectile strength was assessed using the Erectile Hardness Scale.^15^ Studies have indicated that the time taken to return to penile baseline temperature in naked measurements is delayed by ~30 s compared to penile rigidity.^10^ Anticipating an increased delay due to insulation by blankets and clothing, in‐house experiments on hand skin temperature demonstrated that the time needed to return to baseline temperature following a 1.5*°*C increase rose from 4.9 min in naked conditions to 33.6 min under overnight conditions. As the anticipated increase in penile temperature with clothing and blankets is below 1.5*°*C, the measurement duration for the cooling phase was set at 30 min for the blanket measurements and 10 min for the naked conditions.

### 2.5 Data processing and variable extraction

Temperature data collected with the PicoScope 6 software were extracted to MATLAB R2019a. The Ohmeda 3P T3312 sensor calibration curve was utilized to convert voltage values into temperature values.^13^ A low‐pass filter with a cut‐off frequency of 5 Hz was applied to the signal to eliminate high‐frequency noise. MATLAB was used to plot the data from the annotation sensor, facilitating the extraction of the precise start‐ and endpoint of nocturnal erections, as well as their durations, through the “ginput”’ tool. Four primary outcome variables were defined for penile temperature during the erectile periods, as manually annotated:

- *T*_*peak*_: the highest temperature during the erectile period.
- *T*_*baseline*_: the lowest temperature in the 5 min preceding the erectile period.
- ∆*T* = *T*_*peak*_ *–T*_*baseline*_
- Cooling phase duration: the time between the end of the erection and the return of penile temperature to *T*_*baseline*_.

For statistical analysis, the aforementioned variables and the demographic data of the test subjects were processed using IBM SPSS Statistics 25. The data were assessed for normal distribution using the Shapiro–Wilk test. Significant differences between population characteristics in the randomization groups were evaluated using the Mann–Whitney U test. Descriptive statistics, including the mean (SD) or median (IQR), depending on the distribution of the data as determined by the Shapiro–Wilk test, were utilized to analyse temperature outcomes and test subjects' demographic data. Temperature data were examined for significant differences between baseline and peak temperature (*∆T*) using the Wilcoxon signed rank test. The threshold for statistical significance was set at *α* < 0.05. In instances where missing data were detected during measurements, the aroused erection was repeated. If missing data were identified after measurements, the test subject was excluded from the analysis.

## 3 RESULTS

Ten healthy men participated in the study, with a mean age of 22.1 years (SD: 1.97) and an average BMI of 22.00 kg/m^2^ (SD: 2.44). The median IIEF‐5 score was 24.00 (IQR: 2.00). None of the participants had a history of smoking or any other relevant medical history. Participant characteristics are summarized in Table 2. One participant was excluded from the study due to non‐interpretable results resulting from incorrect placement of the data logging system on the thigh, which affected the penile temperature measurements.

**TABLE 2:** Overview of the subject characteristics. Outcomes are given in mean (SD) or median (IQR) dependent on normality.

	Total	Group A	Group B	*p*‐Value
No. of subjects	9	4	5	
Age (years)	22.1 (SD: 1.97)	22.75 (SD: 2.22)	21.6 (SD: 1.82)	0.453
IIEF‐5 score	24.0 (IQR: 2.00)	25.0 (IQR: 1.00)	23.8 (SD: 1.30)	0.281
BMI (kg/m^2^)	22.0 (SD: 2.44)	20.3 (SD: 2.23)	23.3 (SD: 1.82)	0.111

When comparing the randomization groups, no statistically significant differences were evident in age, IIEF‐5 score or BMI. Furthermore, comparison between groups revealed no significant disparities in baseline temperature for both naked and blanket measurements (*p* = 0.556 and *p* = 0.286). Similarly, there were no significant differences in naked peak temperature or erection strength (*p* = 0.556 and *p* = 0.063, respectively). Six erectile phases exceeded the expected duration of 10 min, with five of them occurring in the test subjects from Group B. As a result, the duration of the erectile phases was significantly shorter in Group A (*p* = 0.043) with an average duration of 7.47 min (SD: 1.89), in comparison to 9.84 min (SD: 2.92) in Group B.

The mean duration of the erections was 8.78 min (SD: 2.73), with a median strength of 4.00 (IQR: 1.00) on the Erection Hardness Scale. When comparing the naked measurements with the blanket measurements across all test subjects, there were no significant differences observed in erection duration and strength.

**FIGURE 2:**
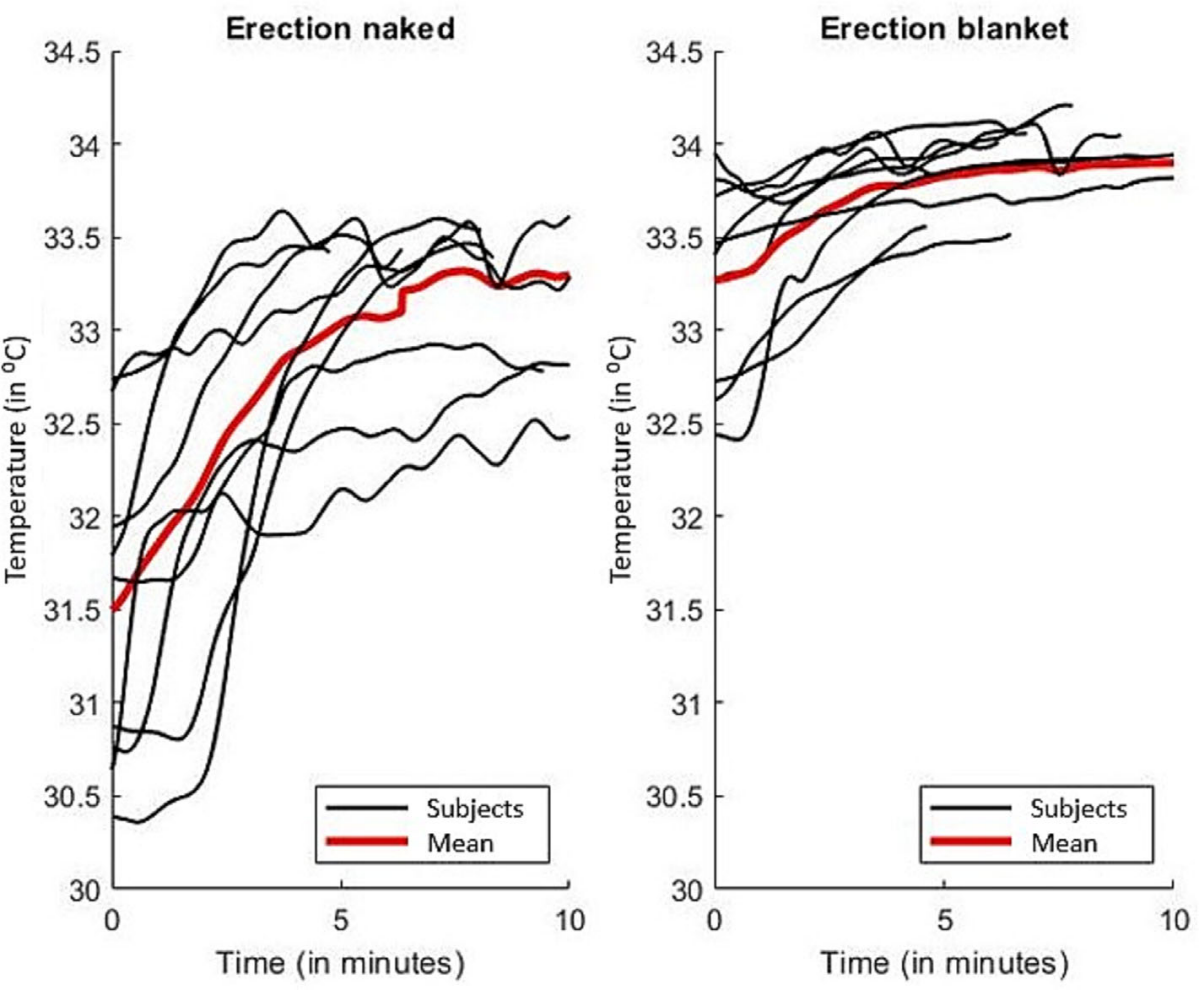
Overview of the penile temperature during visually aroused erection in naked (left) and blanket (right) measurements. The individual courses are shown in black and mean penile temperature in red.

Table 3 presents the outcomes of study variables for both measurement conditions, as depicted in Figure 2. This figure illustrates penile temperature changes during both naked and blanket measurements. In the naked measurements, the mean penile baseline temperature was 31.6*°*C and significantly (*p* < 0.01) increased to 33.3*°*C during erection. Conversely, for the blanket measurements, the baseline temperature was 33.2*°*C and increased significantly (*p* < 0.01) to 33.9*°*C during erection. The increase in penile temperature ranged from 0.26 to 1.55*°*C. The penile temperature increase was significantly lower in the blanket measurements than in the naked measurements (*p* < 0.01). Furthermore, both baseline temperatures and peak temperatures exhibited significant differences between the two conditions (*p* < 0.01).

**TABLE 3:** Overview of the outcomes of the penile temperature measurements for the naked and blanket measurements (*n* = 9). Values are given in mean (SD). T_baseline_ is the minimal temperature in the 5 min prior to erection, T_peak_ the maximal temperature during erection and ΔT the difference between T_baseline_ and T_peak_ during erection.

Variables	Naked	Blanket	*p*‐Value
*T*_*baseline*_ (in ^*°*^C)	31.56 (SD: 0.97)	33.25 (SD: 0.55)	0.004
*T*_*peak*_ (in ^*°*^C)	33.26 (SD: 0.47)	33.91 (SD: 0.24)	0.008
∆T (in ^*°*^C)	1.70 (SD: 0.65)	0.67 (SD: 0.41)	0.004

**FIGURE 3:**
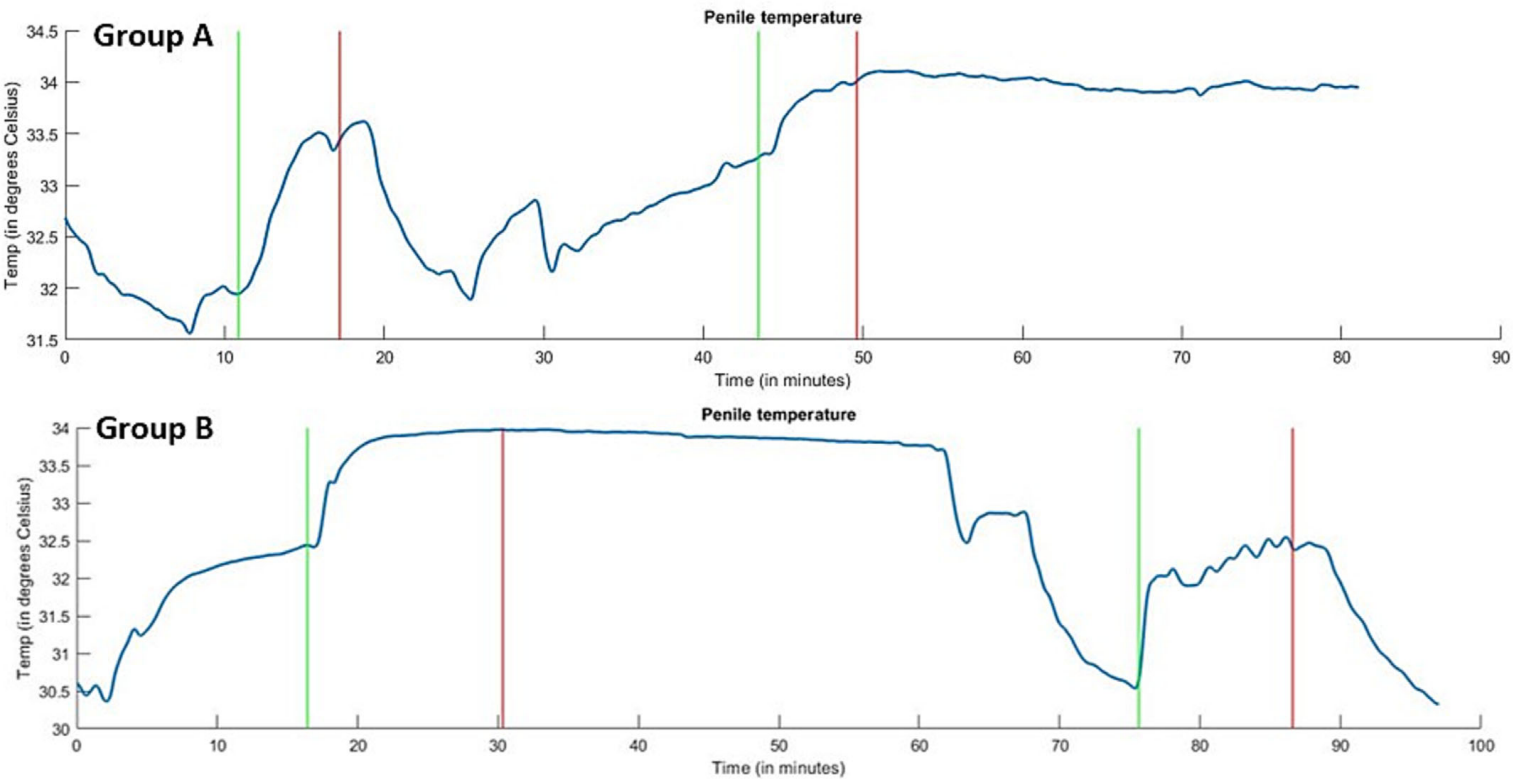
Penile temperature measurements of two subjects. The top image shows outcomes of a test subject in randomization Group A and the bottom image of Group B. The start and end of erection are marked with, respectively, green and red lines.

Figure 3 presents complete penile temperature measurements of two participants, one from Group A and one from Group B, to visualize penile temperature patterns. In naked measurements for all nine participants, penile temperature reached peak levels within 3 min. Following erection, there was a rapid decline in penile skin temperature, returning to baseline within an average of 6.83 min. In the blanket measurements, a similar initial increase in penile temperature was observed at the onset of the erections. This increase was evident in a noticeable alteration in the penile temperature slope. Figure 3 illustrates this slope change occurring at 43 min for the patient in Group A and at 17 min for the patient in Group B. Comparable slope patterns were observed in seven test subjects. In the two remaining subjects, the penile temperature had not reached the plateau level during the baseline measurements, which made the slope change less visually discernible.

Whereas the penile temperature returned to baseline temperature within 7 min in the naked measurements, the penile temperature did not return to baseline during the 30‐min cooling phase in the blanket condition. Remarkably, there was an average decrease of only 0.11°C during the 30‐min cooling phase. The temperature patterns of all test subjects exhibited this ‘Staying Hot effect’, characterized by elevated penile temperature persisting at the peak levels throughout the entire measurement period following visually aroused erections.

## 4 DISCUSSION

Currently, there is an urgent demand for a modernized alternative to the RigiScan, primarily due to concerns about its patient‐friendliness and the limited clinical application. The ‘Staying Hot’ study represents a pioneering proof‐of‐concept study into an alternative method for detecting erections. The study has demonstrated a significant increase in penile temperature during visually induced erections in simulated overnight conditions, thus indicating the feasibility of using temperature measurements for nocturnal erection detection. Additionally, the controlled measurements conducted in this study have shed light on a novel phenomenon: the ‘Staying Hot effect’, where penile temperature remains elevated at peak levels following an erection in the presence of blankets.

Decades ago, researchers observed a significant increase in the penile temperature during visually aroused erections in naked condition. The temperature increase observed in this study (1.70*°*C in naked measurements) aligns with values reported in previous literature (ranging from 0.6–2.4*°*C).^8^, ^9^, ^16^ Furthermore, the baseline and peak penile temperatures (32.12 and 33.89*°*C, respectively) correspond to values observed in prior studies, thus confirming the validity of the employed sensor system.^8^

The significant difference in baseline temperature between the naked and blanket measurements highlights the thermally insulating effect of blankets and underwear, leading to increased bodily temperature. The elevated T_baseline_ results in a reduced temperature differential between the penile temperature and the surrounding air temperature, consequently decreasing heat flux during an erection. Nonetheless, the temperature increase remains significant, advocating for the feasibility of nocturnal erection detection through overnight penile temperature measurements.

As outlined in the methods section, it was hypothesized that the cooling phase duration would increase under simulated overnight conditions. Unexpectedly, the study revealed an intriguing phenomenon termed the ‘Staying Hot effect’, where penile temperature remained elevated at peak levels. Given that penile temperature decreased immediately after the naked erections and considering that the participants remained in bed during the study, the ‘Staying Hot effect’ can be attributed to the low thermal conductivity of blankets and clothing.^17^ However, the persistent significant temperate increase observed in the simulated overnight conditions supports the feasibility of utilizing penile temperature measurements for nocturnal erection detection. Overnight measurements are likely to yield comparable results, with successive elevated penile temperatures following erections. This may pose a challenge in detecting subsequent nocturnal erections during overnight measurements. Thus, further investigation into the impact of the ‘Staying Hot effect’ on the validity of nocturnal erection detection using the temperature methodology is warranted.

## 5 LIMITATIONS AND RECOMMENDATIONS

The unexpected phenomenon of elevated penile temperature not returning to baseline before the start of the naked measurements in Group B test subjects was not foreseen prior to the study, and thus not addressed in the study procedure. This can be observed in Figure 3. Nevertheless, the validity of comparisons between the randomization groups is supported by the absence of significant differences in baseline temperature between the naked and blanket measurements in these groups. Figure 3 further demonstrates that the body temperature of both test subjects had not yet reached a plateau level of T_baseline_ at the beginning of the erection phase in the blanket measurement. The visible change in the course of the penile temperature at the start of the erection phase confirms that the observed increase in penile temperature is attributable to erectile heat flux. Consequently, the impact of the ‘Staying Hot effect’ on the study's outcomes and conclusions is expected to be non‐significant.

During the establishment of the study, application of the RigiScan within this study for erection annotation was waived as increasing the excitement during study participation could risk the validity of the outcomes. Therefore, erection timing and duration was annotated using an additional temperature sensor placed in the test subjects' hand. Prior studies have identified a correlation between sexual arousal and genital arousal in healthy men, bolstering the validity of the study procedure.^8^ The consistent course of penile temperature during visually aroused erections across test subjects further supports the validity of this annotation method. Furthermore, the similarity in penile temperature courses during visually aroused erections among test subjects indicates that any differences between annotated and actual erectile phase durations are likely to have a minimal impact on the study's conclusions.

To assess the capability of detecting erections under overnight conditions using temperature‐based methodology, the ‘Staying Hot’ study was designed to focus on a cohort of healthy young men. It is essential to acknowledge that the prevalence of ED in men tends to rise with age. A population definition study conducted by Saigal et al.^21^ revealed that 77.5% of men over the age of 75 years are affected by ED. Furthermore, comorbidities such as diabetes mellitus, obesity, cardiovascular disease, hypertension and smoking are associated with an elevated risk for developing ED. In order to validate the applicability of this sensor for the patient population encountered in clinical practice, it is imperative to conduct further investigations into penile temperature during erections in both older healthy men and patients with ED. These studies will provide valuable insights into the effectiveness of the temperature methodology as a patient‐friendly and non‐invasive approach to diagnosing ED.

The data of this study was collected through measurements of visually aroused erections, which result from different physiological processes compared to nocturnal erections.^19^ Nocturnal erections typically last an average of 25 min,^20^ whereas the recorded erection duration in this study was 8.78 min, falling short of the intended 10‐min duration stated in the literature as the time required to reach peak temperature during an erection.^8^ Test subjects reported that the pressure to achieve a visually aroused erection in combination with the excitement about participating in the study led to difficulties in adhering to the anticipated erection duration. However, even with an extended duration, it is likely that the increased duration would have only further demonstrated the feasibility of erection detection using the temperature methodology. Therefore, the influence of reduced erection duration on the study's outcomes is considered negligible.

For five out of the nine test subjects, the penile temperature did not reach a plateau level during the erectile phase. As nocturnal erections typically last ~25 min on average, it is anticipated that the increase in penile temperature during overnight measurements would exceed 0.6*°*C. Nocturnal erections predominantly occur during REM‐sleep, characterized by the complete atonia of voluntary muscles. Consequently, kinetic or friction‐induced temperature changes do not influence penile temperature during nocturnal erections. On the other hand, changes in penile temperature during non‐REM‐sleep stages are susceptible to kinetic or friction energy due to movement. A study by Gunner et al.^18^ demonstrated that rubbing hands together for 10 s results in an increase in hand temperature of 0.4 to 0.6*°*C. This action likely generates more energy than any movement during sleep. Therefore, it is expected that movement‐induced temperature increases in the penis will be lower than the temperature increase during nocturnal erections. Further research is required to determine the actual extent of the increase in penile temperature and its visual detectability during nocturnal erections.

The influence of the ‘Staying Hot effect’ on the detection of nocturnal erections warrants further investigation. Nocturnal erections typically exhibit an average gap of 60 min between occurrences.^22^ The ‘Staying Hot effect’ reveals that, underneath blankets, penile heat can solely conduct. Dissipation of heat through convection is absent. The low thermal conductivity of the blankets resulted in an average temperature decrease of 0.1°C. While cooling phases of 60 min were not observed, it is expected that penile temperature will remain elevated. Given that nocturnal erections take place during REM‐sleep, the ‘Staying Hot effect’ is likely to manifest between successive nocturnal erections. Only airflow passing through the blanket, inducing convection (e.g. due to movement), would result in a decrease in penile temperature. The ‘Staying Hot effect’ could impose a challenge in the detection of subsequent nocturnal erection periods during ambulatory measurements. Conducting an overnight study is essential to determine the feasibility of detecting (subsequent) nocturnal erections using the temperature methodology.

Conducting an overnight study with simultaneous recording of RigiScan data would allow for a direct comparison of the specificity and sensitivity of penile temperature measurements with the RigiScan. This comparative analysis would provide additional insights into the feasibility of nocturnal erection detection using the temperature methodology. This overnight study serves as a pivotal step in validating penile temperature measurements as a valuable methodology to be incorporated into the development of a modernized, patient‐friendly diagnostic tool for ED. This tool not only holds the potential to reintroduce non‐invasive differentiation techniques in ED diagnostics but could also offer value in researching the physiology of erectile (dys)function in women and/or patients with prostate cancer following prostatectomy.

## 6 CONCLUSION

The outcomes of the proof‐of‐concept ‘Staying Hot’ study has provided crucial insights into the influence of overnight conditions on penile temperature during erection. The study revealed that penile temperature significantly increases not only during visually aroused erections in naked conditions but also under a blanket with underwear, with an average temperature increase of 0.67°C. This finding underscores the feasibility of using temperature measurements for (nocturnal) erection detection. Controlled measurements conducted in this study have shed light on the ‘Staying Hot effect’, characterized by the penile temperature remaining at peak levels for over 30 min after an erection in simulated overnight conditions. It has been determined that the ‘Staying Hot effect’ is a result of the presence of blankets and underwear. To further investigate the feasibility and clinical validity of nocturnal erection detection using the temperature methodology, data from overnight penile temperature measurements is essential. However, the results of this study strongly indicate that the methodology has the potential to serve as a promising alternative to RigiScan diagnostics, offering a modernized approach to ED diagnostics.

## AUTHOR CONTRIBUTIONS

All authors have approved the final version of this manuscript.Hille Torenvlied conceived and designed the work that led to the submission. Furthermore, she acquired the data, interpreted the results, and drafted and revised the manuscript. She is accountable for all aspects of the work in ensuring that questions related to the accuracy or integrity of any part of the work are appropriately investigated and resolved. Evelien Trip collaborated in the result interpretation and revised the manuscript. Dr.ir. Wouter Olthuis collaborated in the design of the work that led to the submission. He supported the result interpretation and revised the manuscript. Prof.Dr.ir. Loes Segerink revised the manuscript. Dr. Jack Beck supported the design of the work that led to the submission. Furthermore, he collaborated in the result interpretation and revised the manuscript.

## CONFLICT OF INTEREST STATEMENT

There are no conflicts of interest associated with this study. This study was funded by the St. Antonius Ziekenhuis and University of Twente as part of an academic dissertation study. None of these organizations may gain or lose financially through this publication. The funding source had no further involvement in the study. No financial benefits or support was received from the distributors of the components of the penile temperature measurement system. There is no (desired) patent holding or stock ownership that could cause conflict of interest.
